# Headache and Its Varieties

**Published:** 1854-06

**Authors:** Patrick J. Murphy


Headache and its Varieties.—By Patrick J. Murphy, M. D. —How often in practice do we feel the truth of an observation of the late Dr. Graves, that "no cases prove more troublesome to the practitioner, and for none is he more frequently consulted, than the headaches of young women." It is now some years since I read a paper before one of our medical societies, giving a slight sketch of the symptoms and treatment of the more prevalent forms. Daily observation has confirmed my belief that the doctrine then advanced is in the main correct. As an abstract of the paper appeared in the columns of one of our weekly periodicals, and as it was considered worthy of being re-published in Ranking's *Half-yearly Abstract*, I should not have been tempted to enlarge upon the subject, but for a remarkable sentence in the second volume of Graves' "Clinical Lectures on the Practice of Medicine," p. 552, where, speaking of dry cupping, he observes:—"I have in a previous lecture referred to its effects on the headaches of young ladies. Now these are varied and numerous beyond conception, generally connected with some menstrual irregularity and derangement of the intestinal canal, and forming a class of disorders which would require a good monograph, more than any other I know of. Many practitioners get into disgrace with ladies on this account, and, as a natural consequence, with the community in general."

The cause and seat of the more ordinary headaches, especially in young ladies, are, according to my experience, by no means so obscure, and it tends to confirm the correctness of my views, that the treatment adopted has seldom failed to afford relief.

Headache is one of those complaints for which we are almost daily consulted. If an aged person be the sufferer, the patient becomes alarmed, and the practitioner is kept in a continual state of anxiety while it exists; and if it arises in childhood, and is persistent, it is dreaded as an insidious symptom of hydrencephalus. The discrimination of one form from another seems still difficult, and the treatment is consequently confused, unsatisfactory, and therefore in many cases injurious. With our present knowledge of anatomy and physiology, the pathology of headache should be more exact. The fact is, practitioners have been misled by the multitudinous causes to which headache has been attributed; and hence if there be a temporary functional derangement of a viscus co-existing with headache, the disorder of the head is believed to be sympathetic of the viscus, and the treatment, to use the mildest term, is inefficacious. That there are varieties of headache, it is true; but that those which we are called upon usually to relieve are much varied, is not the fact. In nine cases out of ten the extraordinary and rare cases of headache are easily recognised. Take, for example, the headache originating from fungus hæmatodes, or from scrofulous tubercle in the brain; in the former, incipient amaurosis rouses our suspicion; in the latter, other scrofulous affections always co-exist; and in the last case of the kind I saw, phthisis had already commenced.

We have several classifications of headache. The best is that which divides them into the bilious, the nervous, the rheumatic, the gouty, &c. Yet how defective is the classification? I have never yet met a physician who could define what bilious meant, and least of all a bilious headache, although the term is stereotyped. To lessen this confusion, and to check the unphilosophical mode of employing medical terms which convey only confused ideas, is my apology for attempting a classification, and this classification shall include only the ordinary forms of headaches, for the extraordinary forms neither admit nor require to be classified. The classification which follows is grounded solely on experience, and is limited to fire varieties:—the anæmic, the congestive, the neuralgic, the rheumatic, and the periosteal.

The first two have their source in derangement of the cerebral circulation, and hence the seat is intra-cranial; the other three are extra-cranial. To speak more plainly, two arise from disordered circulation, one from the nerves of the scalp, one from the fibrous structure, and one from the pericranium, which is also a fibrous structure.

The most prevalent form of these headaches, at least in London, is the anæmic, or that which is caused by a deficiency of blood within the cranium. The next, congestive; then the neuralgic, or that identical with spinal irritation; next, the rheumatic, very seldom—a solitary symptom; and lastly the periosteal, almost always traceable to a mercurial course, or to an injury. To this simple classification we may in most cases restrict our attention; and as the three last have peculiar characters revealing themselves on the slightest examination, the whole difficulty of diagnosis is confined, strange as the assertion may sound, to headaches arising from deficiency or excess of blood within the cranium. Circumstances do however occur, which render a diagnosis difficult. The neuralgic may co-exist with the anæmic, or incipient fever will change the anæmic form of this day to the congestive of the next. The latter combination, or rather superseding, is not rare in young ladies, the anæmic form being so common in weak constitutions, and debility predisposing so much to attacks of feverish colds. The doubt, however, is removed in a few days, when the fever declares itself, a correct diagnosis is then obtained, which almost always points out the successful treatment. The periosteal or rheumatic may co-exist with the congestive, and I once saw a puzzling case where the periosteal headache being severe and well-defined, there was also present the anæmic form, the result of profuse menstruation and leucorrhæa. The anemic headache, being the most common and persistent, should be first described.

THE ANÆMIC HEADACHE,

Or headache from deficiency of blood, is the headache of debility. It is an invariable attendant on chlorosis. It is the most common form to which young growing females are subject. Debility arises from various causes:—loss of blood, whether by the lancet, epistaxis, hæmorrhoids, uterine hæmorrhage, &c.; leucorrhœa, prolonged lactation, chronic diarrhœa, profuse perspirations, over-exertions, innutritious diet. It is not unfrequent in young men who indulge too freely in smoking tobacco, nor is it, I regret to say, uncommon in both sexes from masturbation. Debility, the consequence of any of these causes, is attended with headache, and this being frequently the most prominent and distressing symptom, we are consulted for this alone, and any doubts as to the true nature of the headache are easily removed, for, on inquiry we find it co-exists with many, and even occasionally all, of the following symytoms:—

*Symptoms.*—The pain or rather ache is referred to the forehead and vertex, in a line more or less with the sagittal suture; it is usually described as a swimming or vertigo; it is relieved in the recumbent position, and increased by remaining long in the upright posture, or by any additional cause of weakness; no matter how severe it may be before bed-hour, the patient is tolerably free in the morning, just previous to getting out of bed; if the weakness be excessive, there is, on suddenly rising, a tendency to and sometimes actual syncope, or a temporary giddiness compels the sufferer to cling to the bed-post for support, and, as might be surmised, it is occasionally followed by nausea, but actual vomiting is very rare.

*Vision* also suffers, motes or muscæ volitantes dim the sight, and towards night, the feebleness having been increased from the exertions of the day, to some persons, while reading, the letters appear to be so intermingled that the occupation must be deferred for some moments. Complete amaurosis, preceded by headache, has followed the debility of prolonged lactation or hæmorrhage; the face is generally pale, but not necessarily so; the eyes are sunk; there is the dark semicircle beneath the lids; the surface chilly; the feet cold, and, as a consequence, occasional *cramps* in the lower extremities during the night; in very delicate females there is slight œdema of the ankles, for the shoe which fitted easily in the morning is felt too tight at night, if there has been much standing; palpitation of the heart, and breathlessness and faintings on slight exertion; disturbed sleep, attended with nightmare or unpleasant dreams, varied by profound sleep of eight or twelve hours' duration; sinking at the epigastrium; sudden desire for food, which, if not quickly appeased, soon ceases, leaving a languidness; bowels rather constipated; fits of sighing, gaping, or yawning; an unaccountable depression of spirits, which, in young females, is attended with involuntary shedding of tears; disinclination for exertion; easily exhausted by conversation or reading aloud; pulse feeble; lips pallid; tongue flabby, bloodless, expanded, and indented at its edges by the teeth; mammæ cold; nothing positive can be said with regard to menstruation; in chlorosis it is scanty or altogether absent, but menstruation once established, the headache and other symptoms are more often caused by menorrhagia and profuse leucorrhœa; if copious perspirations take place, the debility is more marked, but cramps and a few other symptoms are not then present. In very severe cases there is the venous murmur in the neck, or "*bruit de diable*," œdema of the ankles, and sometimes anasarca of the legs. The *bruit de soufflet* may be sought for when palpitations are excessive.

Numerous and apparently antagonistic as these symptoms are, we occasionally meet them combined, and more or less modified. Inquiring into their origin, excluding the cause of the debility, we find them traceable to the central organ of the circulation. This muscle, in common with the voluntary muscles, is enfeebled from similar causes; when enfeebled it is unable to propel the blood as usual, it becomes what I should call a *flaccid heart*, or, as termed by Dupuytren, to whom this combination of symptoms was familiar, "*rélachement du cœur*."

Admitting the doctrine that this form of headache and its conjoined symptoms are the consequences of the contractile power of the heart being weakened, this explanation is not difficult.

*Headache.*—This variety is known as cephalæa, vertigo, megrim or giddiness. Its history shows that it arises from a deficiency of blood within the cranium. It is relieved in the recumbent, and increased in the erect posture. The sensation may be most annoying on retiring to rest, yet on awaking in the morning it is either altogether gone or very much less. Rest, generous diet, and stimulants oppose its encroachments; exertion, diet small in quantity and bad in quality, great loss of the fluids from diarrhœa, perspiration, lactation, epistaxis or leucorrhœa, add to its intensity. The mind even may become unsound, for it is not uncommon to meet with mothers in the lower classes of life, who, compelled by necessity to toil all day, deprived of rest at night by the cries of their infants, unable to obtain sufficient nourishment, and hourly drained by lactation, whose symptoms, beginning with this variety of headache, are soon preyed upon by low spirits, succeeded by the deepest melancholy; and, if now closely questioned, they will confess they are continually apprehensive of imbruing their hands in the blood of that very offspring for whose welfare they have sapped the foundations of body and of mind. The records of the daily papers unfortunately too well testify that in many pitiable cases I have not overcolored the picture. A suicidal tendency is also an attendant idea in other extreme cases. What an intense headache follows uterine hæmorrhage! the slightest noise is unendurable, and the head cannot be raised from the pillow without inducing syncope.

*Dimness of Vision*, a symptom seldom absent in cases even moderately advanced, as it occurs occasionally only, seldom continues more than a few seconds, and is not very annoying; it will not be alluded to by the patient unless inquired after by the physician. When the headache is very severe, muscæ volitantes are noticed. It is identical with that temporary loss of vision preceding syncope. If the cause be not removed, functional amaurosis may follow, which, if treated by depletives, might be permanent.

*Countenance, lips, and tongue pale.*—No proofs are required that paleness in parts naturally red must arise from a deficiency of blood only.

*The sunken eye and dark semi-circle* beneath the eyelids are well known to denote a languid circulation, as well as some degree of emaciation.

*Coldness of the extremities, surface of body chilly.*—These attendant symptoms, which are always present, corroborate the idea, that in the central organ of circulation resides the primary cause of the headache, and the other symptoms already enumerated. Fear and anxiety depress the action of the heart, and chill the surface of the body. The cold bath, which lessens so speedily the impulse of the heart's action, if prolonged would rapidly abstract caloric from the body, and check its motions for ever. Cool the cutaneous surface, and you lessen the beats of the heart; lessen the beats of the heart by a mental emotion, or by digitalis, and you cool the cutaneous surface. Reverse the medal, and we discover that whatever quickens the circulation diffuses warmth over the surface.

*Palpitation of the heart; feeble pulse; dyspnœa on slight exertion.*—Palpitation is either organic or functional. If the former it is persistent, the pulse is strong and incompressible, and the heart beats with such force against the parietes of the chest as to be sensibly felt by the hand or ear when placed over the cardiac region. These symptoms are reversed when the palpitation is functional and from mere weakness; the pulse is languid and compressible; the heart is quiet during repose, and disturbed only from mental excitement or physical exertion; the impetus is feeble and diffused; and if the person lie in the recumbent position, and the heart be thus withdrawn from immediate contact with the walls of the chest, its beats are scarcely perceptible.

Both the palpitation and dyspnœa are the effects of blood being poured more rapidly into the cavities than the heart in its then weakened state is able to expel. Of course we all know that muscular exertion forces blood to the heart, and therefore there is a demand on the organ for increased action termed palpitation, and not being able to empty its cavities completely and rapidly enough, there is an impediment to the entrance of the blood from the pulmonary veins and consequent pulmonary congestion which induce dyspnœa.

*Cramps*, which are very usual symptoms, have never been suspected to depend on a flaccid heart. A cramp arises when *there is an impediment to the circulation of the blood in a vein which traverses the substance of a muscle.* The heart therefore, when flaccid, not being able to keep up an active circulation, is a cause of cramp. The enlarged uterus, by pressing on the iliac veins, causes cramps in the same way. The severe pain of cramp is owing to the pressure which the muscular nerves endure. Cramps from a flaccid heart are only felt in bed, when the contractions of the muscles of the leg have ceased. The involuntary action of the muscle, although painful, is salutary, for muscular contractions are a great aid to the circulation. The cramps of cholera are also owing to the stagnation of the blood in the muscular veins.

*Disturbed sleep; dreams.*—If during sleep the free action of the heart be impeded, the sleep is broken, disturbed, and attended with unpleasant dreams. A person with a flaccid heart lying asleep on his left side, and thus checking the motion of the ribs, is almost certain to be annoyed with night-mare or unpleasant dreams. It is singular and inexplicable why these dreams should be always of a distressing nature; tumbling down, fire, drowning, or thieves, are the usual subjects. In organic diseases, sleep can scarcely be indulged in, unless in a semi-recumbent position, without the intrusion of these unpleasant dreams. We may therefore conclude, that where headache co-exists with disturbed sleep and disagreeable dreams, we have to combat with a flaccid heart; nor can I recal to memory one well-marked case of chlorosis where those dreams were not acknowledged.

*Sighing, gaping, and yawning*, are closely related to each other; they are natural and necessary acts to relieve præcordial oppression. If the attention be engaged, and the heart not acting strongly, the pulmonary veins are over-distended, and sighing—which is merely taking a deep breath, and calling the diaphragm into full action—becomes necessary to expand the pulmonary tissue.

*Sinking of the epigastrium* is one of the most constant symptoms. This symptom is difficult of explanation, but it seems analogous to the sensation of hunger. Certain it is that food relieves and abstinence increases it. The appetite is craving; but with some persons, although there is great desire for food, yet hunger is easily appeased; and if there be the slightest over-indulgence, distention, tympanitis, and borborygmi, follow. The tympanitis I have usually considered as the result of the muscular coats of the intestines and stomach partaking of the general weakness of the voluntary muscles, which thus yield readily to the gaseous pressure. The muscles, both organic and inorganic, are enfeebled. There is a wish for stimulants even by young females, and also by females who display almost every symptom of chronic gastritis, such as nausea, vomiting, and pyrosis. If, therefore, there be no pain on pressing the epigastrium, and the appetite and wish for stimulants are present, it would be mistaken practice to deplete or give hydrocyanic acid or other sedatives.

*Œdema of the ankles.*—Many diseases produce this symptom, especially those which drain the system. In prolonged chlorosis it is not infrequent, and denotes a flaccid heart unable to exhaust the veins of their blood. To the same cause must be attributed this symptom co-existing with anæmic headache. It does not appear early, sometimes it is denied, although it will be admitted that towards night the shoes are too tight for the feet. In the delicate and relaxed female the plantar fascia readily yields to the effused fluid; but in more advanced age, and especially in those of once rigid fibre, I have been consulted for this symptom alone in the incipient stage; for then there is severe pain along the sole of the foot, which subsides if the recumbent position be adopted for a few hours, returns when the upright posture is re assumed, and, if not properly treated, is not relieved until œdema explains the cause. In connexion with œdema of the ankles may be also mentioned enlargements of the liver and spleen. The increased volume of the liver is a well known effect of organic affections of the heart, especially of adherent pericardium, but has not been noticed, as far as my recollection goes, as a very common effect of a flaccid heart. In debilitated women, who have had a family, it is by no means uncommon to be consulted for a pain in the right hypochondrium; and if the side be examined while she stands, the practitioner is surprised to discover the liver two or three inches below its level. If, as is too often the case, it is prescribed for as chronic hepatitis, the constitution will be injured; for it is merely venous congestion, and the appropriate remedies are chalybeates and quinine.

*Menstruation*, if absent or deficient, will guide the physician when his patient is a female, and warn him against depletion; but on the other hand, a frequent cause of headache is traceable to the debility arising from profuse menstruation and leucorrhœa. The profession are certainly indebted to Dr. Bennet for first pointing out to the practitioners of this country the intimate connexion there is between these symptoms and ulceration of the cervix uteri. But this ulceration or abrasion—for in many cases it is nothing more—must be curable by other means as well as by the application of nitrate of silver, as many unmarried females are restored to health by injections of acetate of zinc and the exhibition of sulphate of quinine acidulated. When the heart becomes flaccid from innutritious diet or over-exertion, there is not sufficient blood to spare for menstruation; and, on the other hand, profuse uterine secretions weaken equally the voluntary and involuntary muscles.

Unfortunately a great difficulty of diagnosis exists in our profession when co existing symptoms arise from different diseases. The anæmic headache may exist for years, and then have the neuralgic superadded, but this is not of so much practical importance, as the remedies for the one form do not make the other worse; on the other hand a delicate female, long suffering from anæmia and its headache, is often attacked with fever, and the anæmic is thus replaced by the congestive headache. If stimulants be now given, serious mischief may be the consequence, while, on the contrary, a practitioner who sees her for the first time may deplete too largely and produce a tedious convalescence. These mistakes can occur only on the invasion of the fever, for in a few days, the thirst, heat of skin, and loaded tongue, point out clearly what is to be contended with. Cupping or leeching will relieve the head for a few hours, but if the fever be ataxic a degree of prostration may be induced from which the patient can never be roused. I have never seen the anæmic and rheumatic headache combined; the combination is rare, but there is no reason why such may not occur.

*Treatment.*—As debility not only attends, but is often the sole cause of this form of headache, the treatment must, of course be directed in accordance with this view. The diet is most important and the proper kind at once suggests itself; it should be nutritious, and as the muscular coats of the stomach and intestinal tube have lost their tone, or, more correctly speaking, have their contractile power weakened, common sense points that it should be easy of digestion. Animal food is indispensable; it may be taken twice a day. Mutton is to be preferred. Beef, unless stewed, lies heavy on the stomach of weak people. The lean of roast pork may be permitted; it is a variety, and digestible. The flesh of young animals is neither as nourishing nor digestible as those of mature age. Wild fowl, hare, or rabit, seldom disagree. Roast meat contains more nutritious matter than boiled, but either may be taken according to the fancy of the patient. The richest soups and strongest jellies are in every way inferior to the meat from which they are produced; even in a healthy stomach they cause flatulence and distension, and, *a fortiori*, the weak stomach cannot escape. The more solid fish, such as sole, turbot, &c., may be permitted. Stewed eels are wholesome and agreeable. Oysters fresh, uncooked, and cut into three or four portions, never disagree. Vegetables, unless potatoes, should be cautiously used. Bread should be stale, nothing is more indigestible than fresh bread or buttered toast. An excellent evening meal can be made with tea and rusks. Of fruits, strawberries, raspberries, gooseberries, pears, peaches, and plums are agreeable and aperient; uncooked apples usually disagree. Nuts, almonds, and raisins frequently give rise to painful feelings in the stomach. There is a craving for stimulants, which ought to be indulged in moderation. Ale or porter may be allowed at dinner and supper; perhaps porter is preferable, as it usually contains a chalybeate. Bitter ale is useless. A glass of wine between breakfast and luncheon, with a biscuit, is always found grateful and invigorating. To alcoholic drinks the objections are self-evident, especially when young females are the patients. Very delicate females are much benefited by breakfasting in bed. The meals should be light, and repeated whenever the faintness or sinking of the stomach is approaching. Many cases, however, will occur, more particularly in young men, where no directions for diet will be needed, almost all kinds of food being digestible.

All causes of exhaustion should be guarded against. There is nothing more injurious to a flaccid heart than smoking, many cases being traceable to this cause alone.

The medicine on which the greatest reliance may be placed is iron. Fortunately, this remedy can be exhibited in various forms and combinations. The formulas in the Pharmacopœia are as numerous as those of mercury. Griffith's mixture is an excellent mode of prescribing iron, but as the myrrh is unpalatable and useless, it may be omitted; or a form for which we are indebted to Mr. Donovan may be more advantageously substituted; it is as follows: pure sulphate of iron, one drachm; magnesia, ten grains; purified sugar, one ounce; rose or cinnamon water, eight ounces; mix. This is a scientific prescription; and if the iron be free from red oxide, the green color is preserved for eight or ten days. The magnesia neutralizes the sulphuric acid, and converts the sulphate into a protoxide. The sugar prevents decomposition, and it may be flavored with mint or peppermint water. In hospital practice it would be found most economical, and treacle might be used instead of sugar. In the hysterical female, infusion of valerian adds to its value; and if there be great sense of exhaustion, ammonia in combination is most beneficial. Persons will take

Pills who object to fluid medicines. The compound iron pill might be improved by using treacle and potash, which keep the pill soft; and my omitting the myrrh, which only adds to the size. If the cause of the debility be from leucorrhœa, preparations, or hæmorrhage, the tincture of sesquichloride of iron, in doses of fifteen drops, three times a day, will be the most certain form to employ at first. Young unmarried females, from about their twenty-second year, are very subject to a chronic gastritis, or rather irritable stomach; for these the best preparation is the carbonate of iron, with sugar, of the Edinburgh Pharmacopœia. If the appetite is bad, sulphate of quinine may be combined with iron. The occasional constipation, which is caused by the loss of tone, the sulphate of zinc, with small doses of sulphate of strychnia, relieves. In severe chlorosis, the crystallized citric acid aids the iron; and in scrofula,iodide of potash mav be joined with the iron mixture. If there be a periodical neuralgia, the most effective form is the precipitate of carbonate of iron. In a severe case of chorea and anæmic headache, Fowler's solution of arsenic was combined with Donovan's mixture, and in fourteen days both diseases were relieved permanently. When there is a tendency to œdema, and there is any objection to the tartrate of iron and potash, a chalybeate in another form may be prescribed, and supertartrate of potash taken as common drink. The bowels should be kept gently open by an electuary of senna or the compound rhubarb pill. Friction over the cutaneous surface is very useful; in cold weather, the hands and feet ought to be well rubbed two or three times a day, to guard against the existence of chilblains, to which there is a great tendency in these constitutions. In a case of polypus growing from the fundus uteri, attended with profuse discharges and anasarca of the lower extremities, chalybeates kept the symptoms in check, and relieved the severe headache, until the protrusion of the tumor permitted the application of a ligature. Minute directions for the treatment, however, are not necessary, for the form of headache being ascertained, the proper remedies are obvious.—*London Lancet.*

